# Changes in expression of VE-cadherin and MMPs in endothelial cells: Implications for angiogenesis

**DOI:** 10.1186/2045-824X-3-6

**Published:** 2011-02-14

**Authors:** Manikantan S Kiran, Raveendran I Viji, Sameer VB Kumar, Athira A Prabhakaran, Perumana R Sudhakaran

**Affiliations:** 1 Department of Biochemistry, University of Kerala, Thiruvananthapuram, Kerala-695581, India; 2 Department of Biochemistry and Molecular Biology, School of Biological Sciences, Central University of Kerala, Kerala-671328, India; 3 Health Research Institute, National Institute of Advanced Industrial Science and Technology, AIST Shikoku, Takamatsu, Kagawa-761-0395, Japan

## Abstract

The mechanism of cell-cell contact dependent regulation of pericellular proteolysis in angiogenesis was examined by studying the expression of MMPs using isolated HUVECs in culture. Zymography, Immunoblot and RT-PCR analysis showed that the production and secretion of matrixmetalloproteinase-2 and matrixmetalloproteinase-9 by HUVECs in culture were high when they remain as individual cells and significantly decreased during later stages of culture when cells developed cell-cell contact and tubular network-like structure. As MMPs decreased there was significant upregulation of VE-cadherin in cells undergoing angiogenic transition. Investigations to understand the signaling pathways downstream of VE-cadherin showed a relatively high level of β-catenin in the nucleus of endothelial cells in culture during initial stages and decrease in its levels in the nucleus, associated with an increase in the cytosol during later stages of culture. The distribution of β-catenin was found to be regulated by Tyr/Ser phosphorylation status of this protein. Cell-cell contact dependent downregulation of MMPs during angiogenesis was also observed in experiments using proangiogenic substances which caused a rapid rate of downregulation of MMP-2 and MMP-9 and absence of downregulation of MMPs when treated with anti-angiogenic agents.

## Introduction

The ability of ECs (endothelial cells) and their supporting cellular elements to modify their immediate surrounding extracellular matrix (ECM) responding to multiple signals received from the environment is critically important during angiogenesis [1,2]. Both cell-cell and cell-matrix interactions are crucial in the transition of endothelial cell phenotype associated with angiogenesis [3,4]. These interactions are governed by changes in both cell surface receptor for matrix proteins and the nature of the ECM. Matrixmetalloproteinases (MMPs), by virtue of their ability to degrade components of the ECM can influence these processes by altering the composition and structural organization of the ECM, thereby altering matrix-derived signals [1]. Tight regulation of the activity of MMP is required during angiogenesis as excessive proteolysis can cause unwanted damage to the cells and might dissolve the matrix, cell adhesion molecules and receptors needed for anchoring the migrating cells and for the maturation of the neo-vessel [1,5].

A temporal relation between the production of MMPs and the onset of angiogenesis has been reported [6]. Down regulation of MMP-2 and MMP-9 under conditions where human umbilical vein endothelial cells (HUVECs) undergo morphological changes to form tubular network-like structure and higher levels of these enzymes under conditions where cell-cell contact was less and formation of such structures did not occur, suggest that down regulation of MMP production by endothelial cells is crucial to angiogenic process [6]. Further, MMPs have been reported to alter with change in endothelial cell shape where maximum activity was reported when the cells were spherical in shape [7].

Apart from MMPs, cell adhesion molecules are also equally important regulators of angiogenesis. They form intercellular junctions between endothelial cells, which give the endothelium the ability to control the passage of solutes and circulating cells [8] and endothelial surface polarity [9]. They regulate the initiation and maturation of newly formed vessels during angiogenesis. The initiation of angiogenesis occurs when the continuity of the endothelial layer is interrupted due to the loosening of the cell-cell contacts enabling the endothelial cells to proliferate and migrate to the free area [10,11]. During the later stages of angiogenesis (maturation phase) it is essential that the endothelial cells establish the intercellular contacts in order to maintain the morphological integrity and quiescence of the newly formed vessel [12,13]. It thus appears that the molecular mechanisms that govern formation and stabilization of cell-cell contact and pericellular proteolysis are suitably coordinated and regulated. Recent efforts to understand the functional link between cell-cell adhesion and pericellular proteolysis provided data in support of a role for E-cadherin (epithelial-cadherin) in the regulation of expression of MMPs in epithelial cells [14]. Lower expression of MMPs in cancer cells overexpressing E-cadherin and upregulation of MMPs associated with downregulation of E-cadherin expression has been observed [15-17]. Further, blocking cell-cell junction formation in pre-malignant keratinocytes by function blocking antibodies against E-cadherin caused up regulation of MMP-9 [18]. But mechanisms regulating the expression of MMPs in endothelial cells during angiogenesis are still unclear. MMPs have been reported to be regulated at transcriptional, translational and post-translational levels [19]. Nuclear factor kappa B (NFκB) and activator protein-1 (AP-1) signaling pathways have been reported to be the major signaling pathways in the regulation of MMP expression [19]. But it is unlikely that these pathways are involved in the downregulation of MMPs during angiogenesis as significant activation of these signaling pathways as evidenced by the upregulation of a number of genes responsive to these transcription factors occur in ECs during angiogenesis [20,21]. The downregulation of MMPs at later stages of angiogenesis when cell-cell contacts are established and tubular network-like structures are formed tempted us to postulate cell-cell contact dependent mechanisms in the regulation of MMPs. Recent reports indicate that the components of the intercellular adheren junctions also function in intracellular signaling during angiogenesis [22]. VE-cadherin (vascular endothelial-cadherin) has been reported to be the major endothelial specific cell adhesion molecule that plays important role in vascular morphogenesis and growth control [23]. The molecular mechanisms involved in cell-cell contact dependent regulation of MMPs in endothelial cells undergoing angiogenic process was examined using HUVECs in culture and the results show reciprocal changes in the expression of MMP-2 and MMP-9 and VE-cadherin as the cells undergo angiogenic transition.

## Materials and methods

### Materials

MCDB131-medium, gentamycin, antibiotic-antimycotic solution, gelatin, ortho-dianisidine, DEPC, curcumin, ursolic acid, gelatin, FCS, diamino benzidine, Tris, glycine, bovine serum albumin, protein A-sepharose beads, antibodies to MMP-2 (M4065), MMP-9 (M5427), VE-cadherin (C1821), β-catenin (C2206), CD 31 (C7714), CD 14 (C7673), CD 71 (C2063), phospho tyrosine (P5872), phospho serine (P5747), HRP (A6154, A0168) and FITC (F6397) conjugated secondary antibody were purchased from M/s Sigma Aldrich Co (St. Louis, MI). NC membrane was from BioRad laboratories, Hercules, CA. ELISA and tissue culture plates were from NUNC A/S (Roskilde,Denmark). Perfect RNA mini isolation kits and C-Master RT Plus PCR kits were purchased from Eppendorf (Eppendorf AG, Hamburg, Germany).

## Methods

### Isolation and culture of HUVECs

Endothelial cells were isolated by collagenase perfusion of umbilical vein as described before [24]. The yield and viability of isolated HUVECs were determined by trypan blue staining. 1.5 × 10^6 ^cells per well in MCDB 131 medium were seeded in NUNC multi-well plates and allowed to attach for 5 hrs, unattached cells were removed, fresh serum-free medium was added and maintained in culture overnight before starting the experiment. The purity of the cultures was assessed by checking for possible contamination with other cell population like monocyte/macrophage by monitoring expression of CD 14 and CD 71 by immunocytochemical technique. The cultures were negative for both the CD markers. Further, FACS analysis of the cultures showed that about 99.8% cells were CD 31 positive. All the experiments were performed according to the guidelines of the Institutional Ethical Committee.

### Enzyme Linked Immunosorbent Assay (ELISA)

Indirect ELISA was performed using specific antibody [25]. Cell culture medium precoated onto ELISA plates served as the antigen. o-dianisidine was used as substrate and the absorbance of the coloured horse radish peroxidase (HRP) product was measured spectrophotometrically at 405 nm by an automated microplate reader (Thermo Multiskan Sprectrum).

### Reverse Transcriptase-Polymerase Chain Reaction (RT-PCR) analysis

Total RNA was isolated from HUVECs using Perfect RNA Mini isolation kits procured from Eppendorf according to manufacturer's instruction. The primer pairs for human MMP-2, MMP-9, VE-cadherin, β-catenin and GAPDH were as follows; MMP-2 (195 bp) Forward 5'ATGGGGAATCGGTTGAAGG3' Reverse 5'AATTGCATTTCCTGACAGAAGG3'; MMP-9 (147 bp) Forward 5'GACTTGGCAGTGGAGACTGCGGGCA3' Reverse 5'GACCCCACCCC TCCTTGACAGGCAA3'; VE-cadherin (149 bp) Forward 5'GCACCAGTTTGGCCAATATA 3' Reverse 5'GGGTTTTTGCATAATAAGCAGG3'; β-catenin (291 bp); Forward 5'TTTTTAAA GGCAAGAATGCCTCA3' Reverse 5'CATTAATGAAGGCAAGTAGCCCA3' and GAPDH (680 bp) Forward 5'CGGAGTCAACGGATTTGGTCGTAT3' Reverse 5'GCAGGTCAGGTCC ACCACTAGC3'. The primer sequences were taken from NCBI nucleotide database and custom synthesized by Sigma Aldrich Chemicals Bangalore, India. RT-PCR was performed in an Eppendorf thermocycler, using the C-Master RT Plus PCR kit (Eppendorf AG, Hamburg, Germany). 20 μl (2 μg) of the isolated RNA was used as template for reverse transcription and amplification as described before [26].

### Immunoblot Analysis

Medium was removed, cells were washed with phosphate buffered saline (PBS), scraped from the culture plates, centrifuged and the cell pellets were lysed in Laemmli sample buffer. Protein equivalent amount of the lysates were subjected to sodium dodecyl sulfate polyacrylamide gel electrophoresis (SDS-PAGE) on 10% polyacrylamide gels. Proteins were transferred to the nitrocellulose (NC) membrane using a semidry blotting apparatus, probed with specific antibody and developed using HRP-conjugated secondary antibody [27]. Appropriate negative controls were taken without primary antibody. The band intensity was determined by Quantity One 4.5.0 Image acquisition and Analysis software (BioRad).

### Zymography

The activity of MMPs secreted by HUVECs into the medium maintained in culture was determined by gelatin zymography [28]. Zymogram gels consisted of 7.5% polyacrylamide (native) gel polymerized together with gelatin (1 mg/ml). After electrophoresis, the gels were washed twice with 2.5% Triton × 100 and incubated with substrate buffer (50 mM Tris, 5 mM CaCl_2_, pH 7.5) at 37°C for 24 hrs. The gels were stained with Coomassie brilliant blue R 250 and destained with water. Gelatinolytic activities appearing as clear zone were quantitated using Quantity One - 4.5.0 - Software (BioRad).

### Distribution of β-Catenin

Cell pellets were resuspended in 500 μl PBS ice-cold nuclear buffer (150 mM NaCl, 150 mM sucrose, 20 mM HEPES, pH 7.4, 5 mM KCl, 2 mM dithiothreitol, 1 mM MgCl_2_, 0.5 mM CaCl_2_, 0.1 mM PMSF, and protease inhibitors) [29]. The suspension was gently mixed with a pipette on ice for 3 min and then centrifuged at 500 × *g *for 10 min to pellet the nuclei. The cytosolic supernatant was centrifuged for 60 min at 13000 × *g *at 4°C. Protein equivalent amount of cytosolic and nuclear fractions were resolved on SDS polyacrylamide gels and immunoblotted to analyse the levels of β-catenin in nucleus and cytosolic fraction. For immunoprecipitation, equivalent amount of cellular proteins were brought to a volume of 1.0 ml in buffer containing 50 mM Tris-HCl, pH 7.5, 0.4 M NaCl, 5 mM EDTA and 0.5% NP-40. The clear supernatant was incubated with the anti-β-Catenin antibody for 2 hrs on ice followed by Protein A/G-Sepharose for immunoprecipitation. Beads were washed 3 times with the same buffer and extracted in Laemmli sample buffer; subjected to SDS-PAGE, electroblotted and probed with monoclonal antibodies against phosphotyrosine and phosphoserine.

### Statistical Analysis

The statistical significance of difference was analysed by Duncan's one way Analysis of Variance (ANOVA) using SPSS10 Software.

## Results

### Production of matrix metalloproteinases by HUVECs

**Figure 1:**
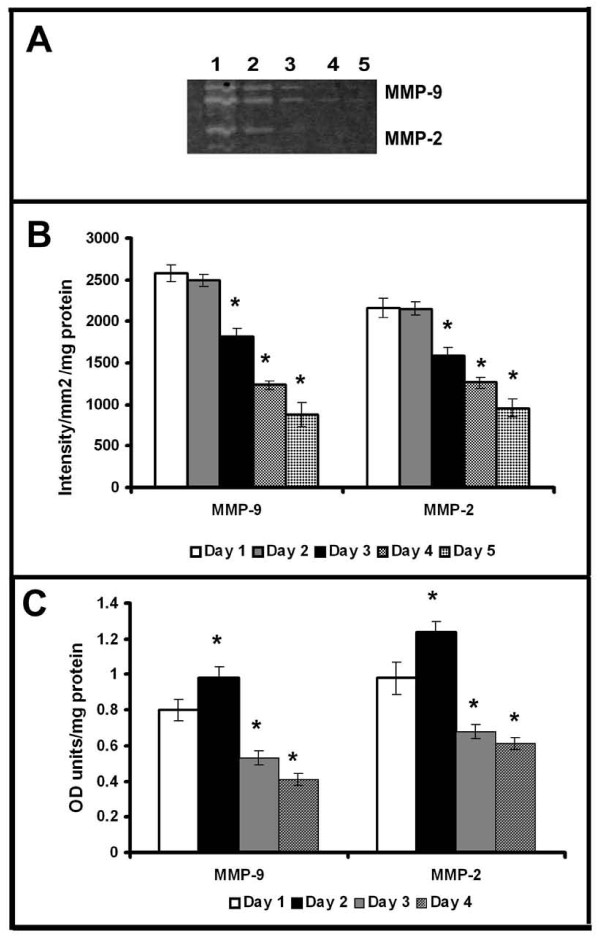
**Production of MMP-2 and MMP-9 by HUVECs**: Equivalent aliquots of the medium normalized to the total cell protein from the cultures of HUVECs maintained in MCDB 131 medium collected at intervals of 24 hrs were subjected to zymography (**A**) and ELISA (**C**) as described in text. Lanes 1-5 1^st^-5^th ^day. The activity of MMP-9 and MMP-2 in zymogram was quantitated using Quantity One 4.5.0 software BioRad geldoc and expressed in arbitrary units of intensity/mm^2^/mg protein **(B)**. Values given are the average of intensity measurement of 6 experiments ± SEM. The protein level expression quantitated for MMP-9 and MMP-2 by ELISA using specific antibody **(C)**. Values given are the average of duplicate analysis of 5 experiments ± SEM. * Statistically significant compared to the production on first day p < 0.05.

**Figure 2:**
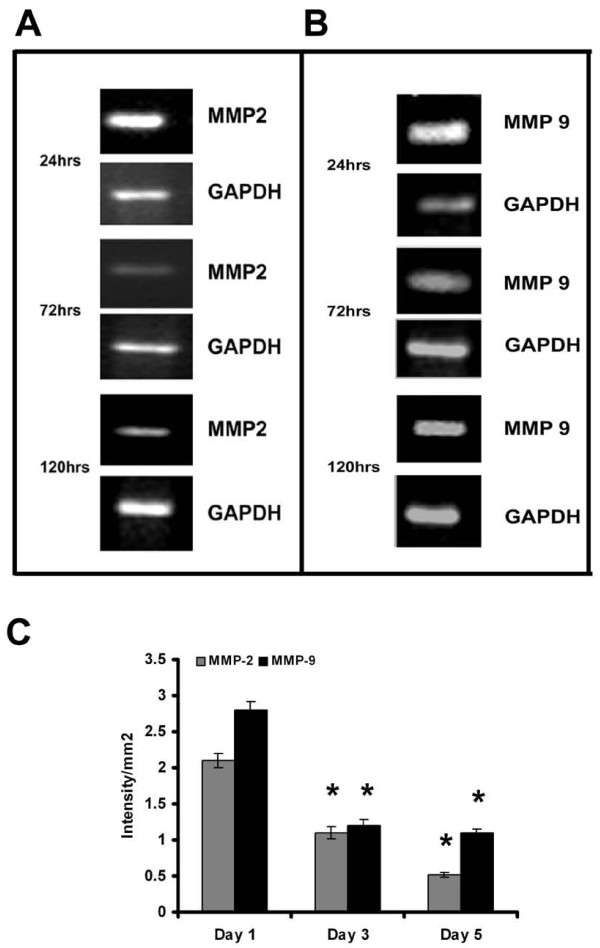
**Expression of MMP-2 and MMP-9 in HUVECs - RT-PCR**: Cells were maintained in MCDB 131 medium for different time intervals. Medium was removed and the cell layer harvested, total RNA was isolated and subjected to RT-PCR as described in the text. Samples were resolved on 1.75% agarose gel MMP-2 (**A**) and MMP-9 (**B**). Intensity of bands were quantitated and normalized with the intensity of band for internal control GAPDH and expressed as intensity/mm2 **(C)**. * Statistically significant when compared to their production on first day p < 0.05.

As reported earlier, with the progression of culture endothelial cell-cell contact, grouping and tubular network-like structures developed [6,20]. In agreement with this data [6], HUVECs in culture in MCDB 131 medium that promoted angiogenic phenotype produced MMP-2 and MMP-9. Zymographic analysis showed a steady decrease in the activity of MMP-2 and MMP-9 with the progression of culture (Figure 1A&1B). Analysis of protein level expression by ELISA (Figure 1C) and western blot (data not given) also showed a decrease in both MMP-2 and MMP-9 which on the 5^th ^day was less than 40% of that on the 1^st ^day. Although there was no significant difference in the activity of MMP-2 and MMP-9 on the 2^nd ^day when compared to that on the 1^st ^day, the levels of the proteins were about 10-15% more on the 2^nd ^day. RT-PCR analysis was done to confirm whether the decrease in the activity of MMP-2 (Figure 2A) and MMP-9 (Figure 2B) was associated with the downregulation of gene expression. The levels of mRNAs of both MMP-2 and MMP-9 significantly decreased with the progression of culture which on the fifth day of culture was less than 30% of that on the first day in cells (Figure 2C).

**Figure 3:**
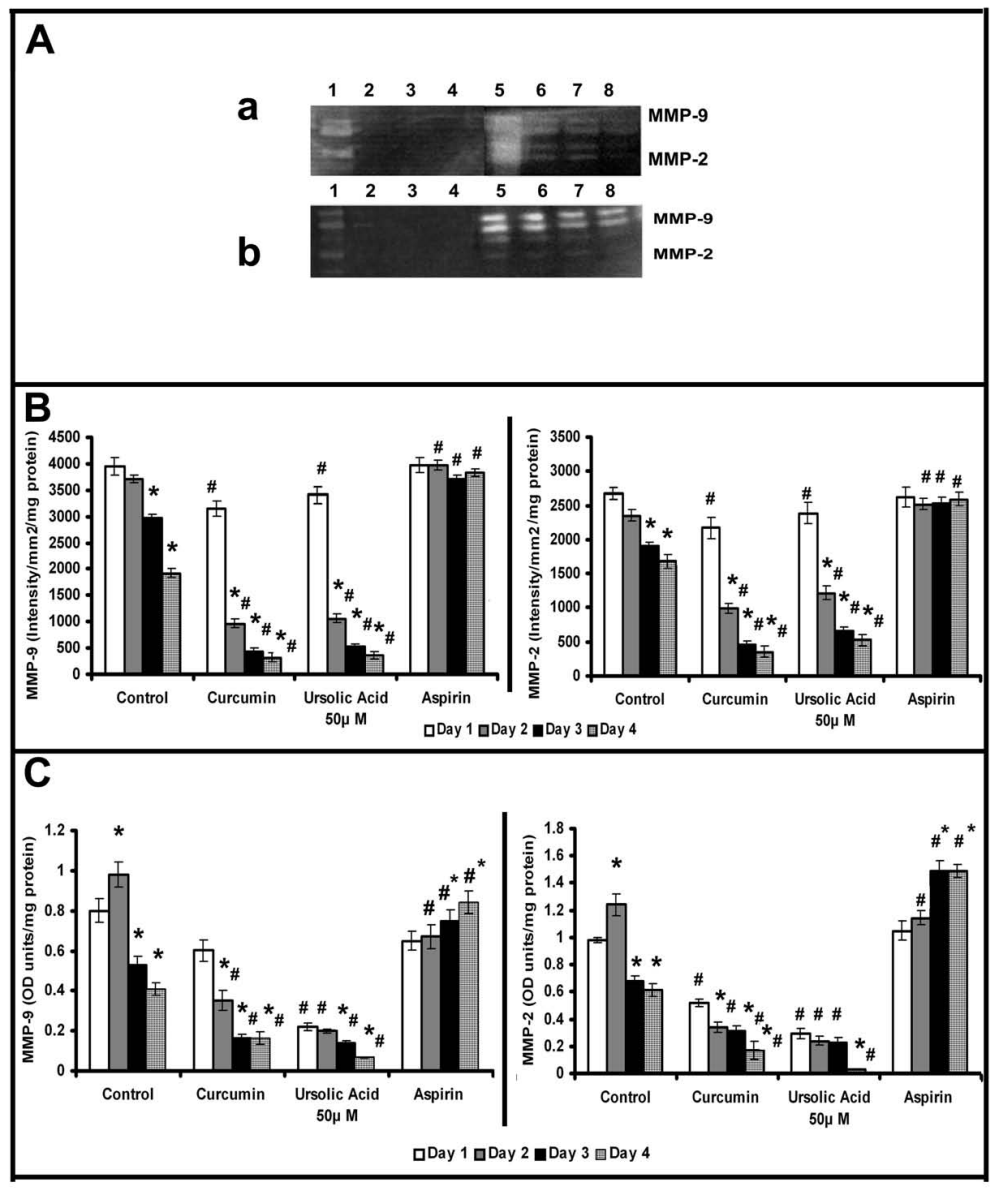
**Effect of modulators of angiogenesis on the activity and production of MMPs**: Equivalent aliquots of the medium normalized to the total cell protein from the culture of control, ursolic acid (50 μM), curcumin (10 μM) and aspirin (100 μM) treated cells on days 1 to 4 were subjected to zymography **(A) **as described in the text.(**a**) Lanes 1-4, 50 μM ursolic acid treated cells 1^st ^-4^th ^day, Lanes 5-8, control 1^st^-4^th ^day. (**b**) Lanes 1-4, 10 μM curcumin treated cells 1^st ^-4^th ^day, Lanes 5-8, aspirin treated cells 1^st^-4^th ^day. The activity of MMP-9 and MMP-2 in zymogram was quantitated using Quantity One 4.5.0 software BioRad geldoc and expressed in arbitrary units of intensity/mm^2^/mg protein **(B)**. Values given are the average of intensity measurement of 6 experiments ± SEM. The culture medium collected from control, ursolic acid (50 μM), curcumin (10 μM) and aspirin (100 μM) treated cells at intervals of 24 hrs (normalized to the total cell protein) were quantitated for MMP-9 and MMP-2 protein expression by ELISA using specific antibody **(**C**)**. Values given are the average of duplicate analysis of 5 experiments ± SEM. * Statistically significant compared to the production on first day (p < 0.05) # Statistically significant compared to controls (p < 0.05).

Inorder to further examine whether the downregulation of MMPs is related with the formation of cell-cell contact and angiogenic transition, experiments were performed with substances which promoted or inhibited cell-cell contact and angiogenic phenotype in HUVECs [20,21]. The downregulation of MMPs associated with development of angiogenic morphology was tested using ursolic acid, a naturally occurring substance that caused proangiogenic effect in HUVECs and accelerated grouping of cells and cell-cell contact formation in serum free MCDB 131 medium [20]. Zymographic analysis showed that ursolic acid caused downregulation of MMP-2 and MMP-9 at a faster rate when compared to untreated controls (Figure 3Aa); ursolic acid at a concentration of 50 μM caused reduction of MMP-2 and MMP-9 to about 40% of that of the initial levels on the 2^nd ^day (Figure 3B). Protein level expression of MMP-2 and MMP-9 also was reduced to about 35% of that of the initial levels on the second day in cells treated with ursolic acid (Figure 3C).

**Figure 4:**
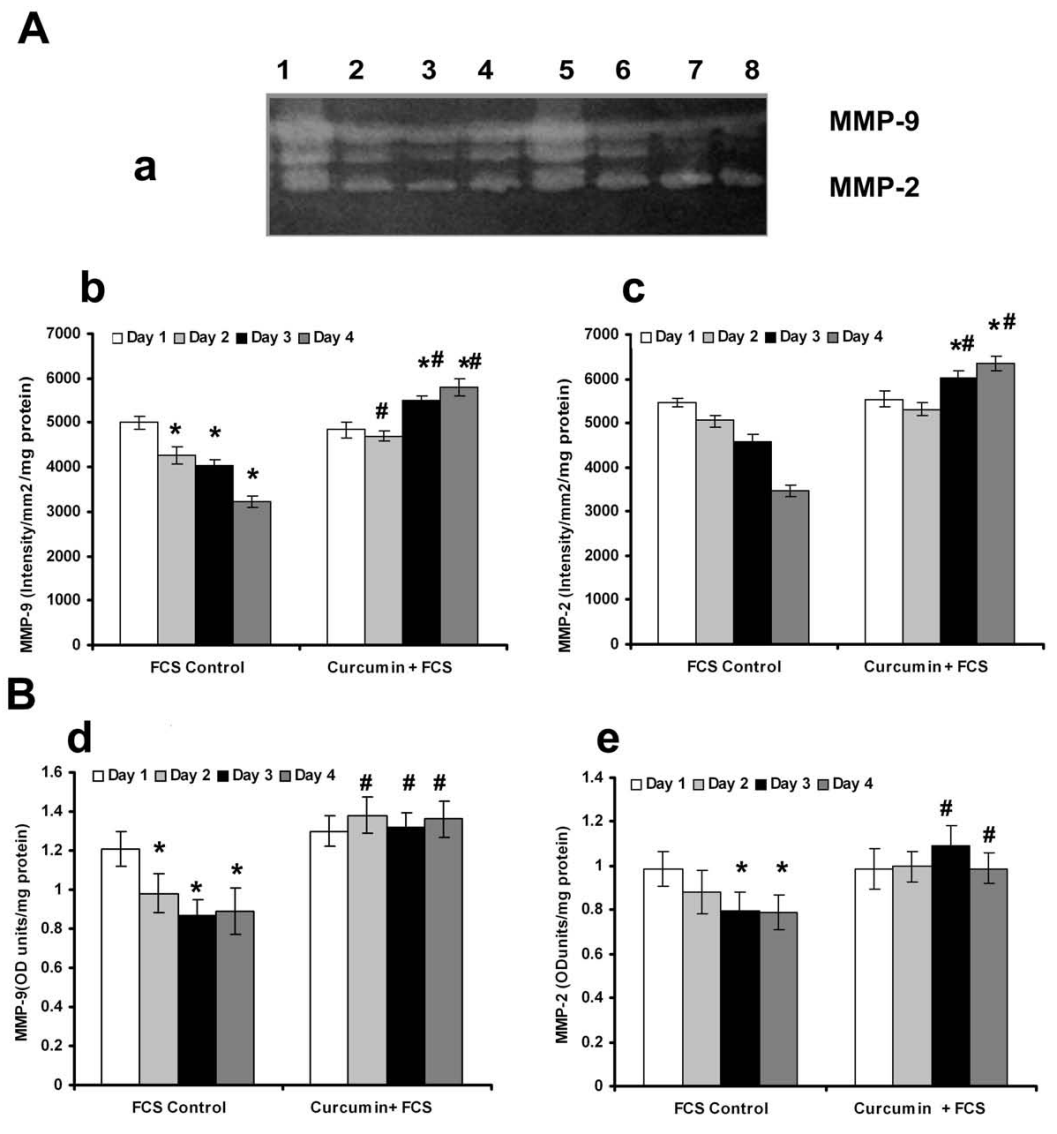
**Effect of supplementation of curcumin in presence of serum on the production and activities of MMP-2 and MMP-9**: (**A**) Equivalent aliquots of the medium appropriately diluted (1:10)from the culture of FCS control and curcumin +FCS treated cells on days 1 to 4 were normalized to the total cell protein were subjected to zymography as described in the text. (**a**) Lane1-4 curcumin + FCS treated cells 1^st ^-4^th ^day, Lane 5-9 control 1^st^-4^th ^day. The activity of MMP-9(**b**) and MMP-2(**c**) expressed in arbitrary units of intensity/mm^2^/mg protein. Values given are the average of intensity measurement of 6 experiments ± SEM. **(B) ELISA **The culture medium collected from controls and curcumin treated cells in presence of serum at intervals of 24 hrs which were normalized to the total cell protein were quantitated for MMP-9(**d**) and MMP-2(**e**) protein expression by ELISA using specific antibody. Values given are the average of duplicate analysis of 5 experiments ± SEM. * Statistically significant when compared to their production on first day p < 0.05. # Statistically significant when compared to control p < 0.05.

It was reported that curcumin caused opposing effects on angiogenesis under different conditions [21]; while in serum free MCDB131 medium it exerted a proangiogenic effect by accelerating the vascular endothelial growth factor (VEGF) mediated signaling, under serum supplemented conditions it exerted anti-angiogenic effect. The changes in MMP activity in HUVECs in response to curcumin were studied in presence and absence of serum. Zymographic analysis showed a steady decrease in the activity of MMP-2 and MMP-9 with the progression of culture in cells treated with curcumin in serum free conditions, the rate of decrease being more than untreated controls (Figure 3A). The activity of MMP-2 and MMP-9 on the second day was less than 25% of that on the first day in cells treated with curcumin whereas in untreated control it was not significantly different from that of 1^st ^day. Analysis of protein level expression by ELISA and western blotting showed that the rate of decrease in both MMP-2 and MMP-9 was more than the untreated control and was related with the angiogenic transition (Figure 3C). Unlike in serum free medium, treatment of HUVECs with curcumin in serum supplemented condition [21], did not cause any downregulation of the activity (Figure 4Aa) or protein level expression (Figure 4Bd&4e) of MMP-2 and MMP-9. These results suggest that the downregulation of MMPs in HUVECs treated with curcumin in the absence of serum, was unlikely to be an effect of curcumin on MMP production, rather it was related with the angiogenic transition of the cells.

Acetyl salicylic acid (aspirin) is reported to inhibit capillary network-like structure formation and angiogenesis [6]. The relation between cell-cell contact formation and MMP production was further tested in HUVECs treated with aspirin which inhibited the formation of cell-cell contact [6]. No downregulation of activity and protein levels of MMP-2 and MMP-9 was observed in cells treated with aspirin (Figure 3Ab).

### Production of VE-cadherin in HUVECs

**Figure 5:**
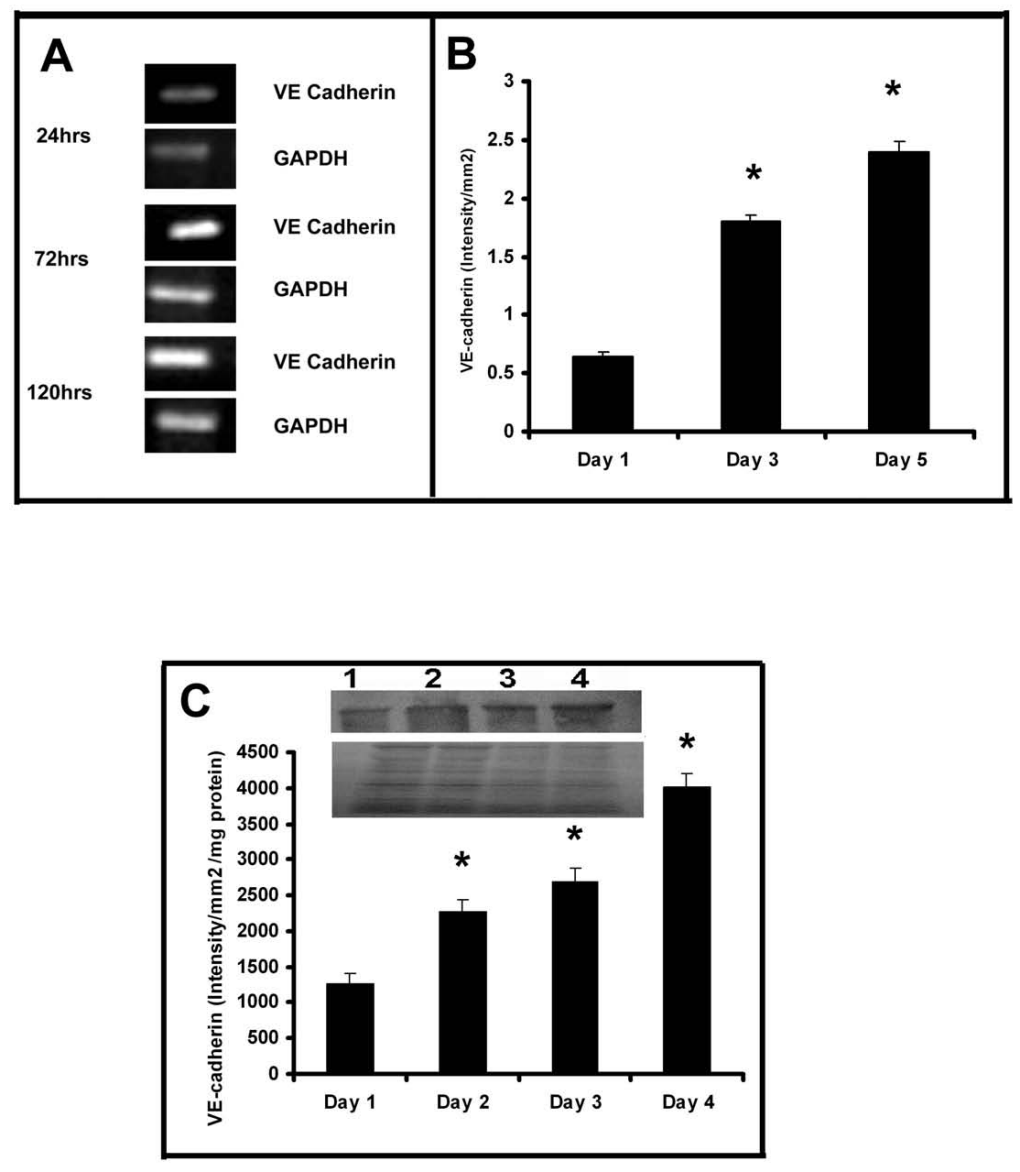
**Production of VE-cadherin in HUVECs (A) RT-PCR**: Cells were maintained in culture in MCDB 131 medium for different time intervals. Total RNA was isolated and subjected to RT-PCR as described in the text. Intensity of bands were quantitated and normalized with the intensity of band for internal control GAPDH and expressed in intensity units/mm^2 ^**(B)**. * Statistically significant when compared to their production on first day p < 0.05. **C. Western blotting **HUVECs were maintained in culture in MCDB 131 medium for different time intervals. Medium was removed, the cells were harvested and lysed in Laemmeli buffer and protein equivalent amount of the cell lysates were subjected to SDS PAGE (bottom) and western blot (top) analysis with anti VE-cadherin antibody as described in the text. Inset, Lane1-4 represents 1^st ^-4^th ^day. The intensity of the immunoblotted bands were measured and expressed in intensity units/mm^2^/mg protein. * Statistically significant when compared to the production on first day p < 0.05.

In order to understand whether there is any relation between the cell-cell contact formation and MMP production, the kinetics of production of VE-cadherin, the intercellular cell adhesion molecule involved in endothelial cell-cell interaction and angiogenesis was analysed by western blotting. There was significant increase in the amount of this protein with the progression of culture which on the fourth day was about 4 fold more than that on the first day (Figure 5C). The expression of VE-cadherin in HUVECs maintained in culture was further confirmed by flow cytometry (data not shown). RT-PCR analysis also showed that the level of mRNA of VE-cadherin progressively increased with the progression of culture (Figure 5A). About four fold increase in the expression of VE-cadherin was observed in cells on the fifth day when compared to its production on the first day of culture indicating an inverse relation between the changes in the expression of VE-cadherin and MMP-2 and MMP-9 (Figure 5B).

### Production and distribution of β-catenin

**Figure 6:**
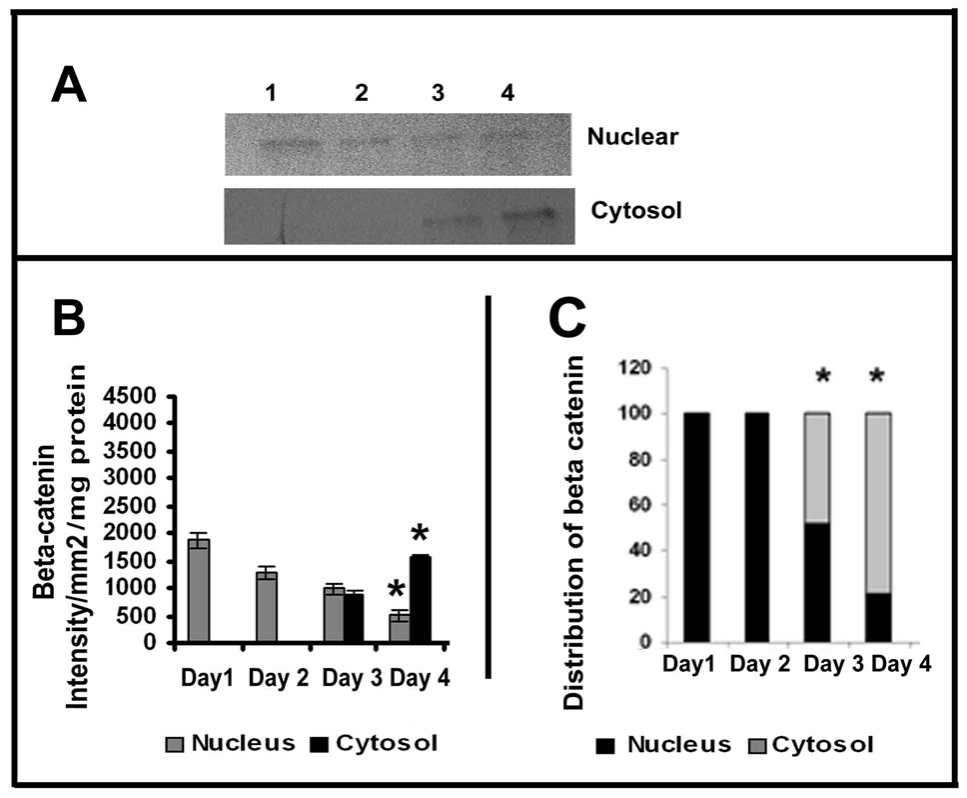
**Changes in nuclear and cytosolic distribution of β-catenin in HUVECs**: HUVECs were maintained in culture for different time intervals as described in the text. Medium was removed, the cells were harvested, fractionated and nuclear and cytosol were isolated. Protein equivalent amount of the cytosolic and nuclear fractions were subjected to SDS PAGE and western blot analysis with anti β-catenin antibody as described in the text. Lane1-4, 1^st ^-4^th ^day **(A)**. The intensity of the immunoblotted bands were measured and expressed in intensity units/mm^2 ^**(B)**. Percentage distribution of β-catenin in the nucleus and cytosol (C) * Statistically significant when compared to their relative levels in nucleus and cytosol on first two days p < 0.05.

β-catenin is one of the accessory proteins associated with the cytoplasmic tail of VE-cadherin aiding the stabilization of cell-cell adhesion. When unbound to VE-cadherin, β-catenin from cytoplasm can translocate into the nucleus. The relative levels of this protein in the cytosol and nucleus were analysed by western blotting and the results are shown in Figure 6. During the initial stages (upto 48 hrs) of culture, almost the entire amount of β-catenin was present in the nucleus, which declined thereafter concomitantly with significant increase in its level in the cytoplasm during later stages of culture (Figure 6A); on the third day only about 50% of the total β-catenin was present in the nucleus, which declined further with the progression of culture (Figure 6B&6C).

**Figure 7:**
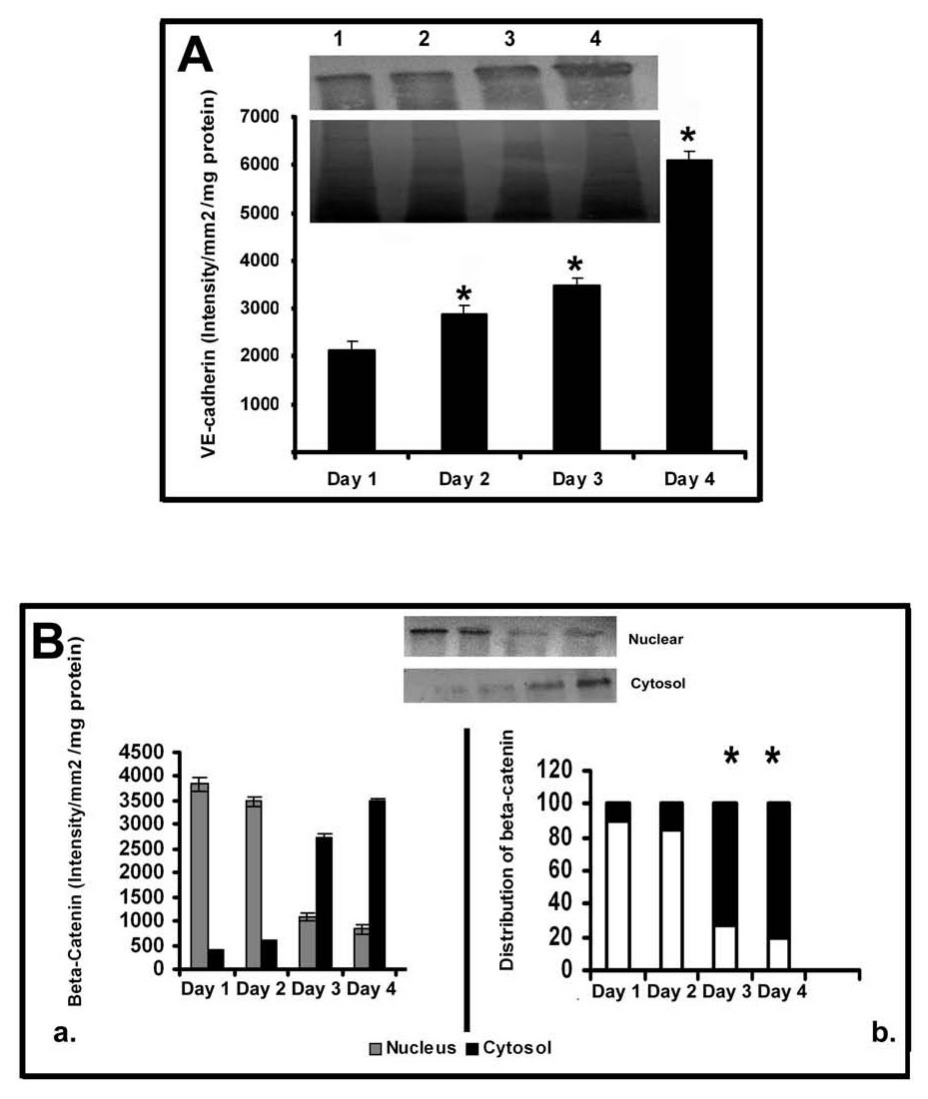
**Changes in the production and distribution of VE Cadherin and β-catenin in HUVECs: Effect of curcumin. (A) Production of VE Cadherin-Western blotting**: HUVECs were maintained in culture in MCDB 131 medium in presence of curcumin (10 μM) for different time intervals. Medium was removed, the cells were harvested and lysed in Laemmeli buffer and protein equivalent amount of the cell lysates normalized to the total cell protein were subjected to SDS PAGE (bottom) and western blot (top) analysis with anti VE-cadherin antibody as described in the text. Lanes 1-4 represents 1^st ^-4^th ^day **(A)**. The intensity of the immunoblotted bands were measured and expressed in intensity units/mm^2^/mg protein. Values for control in intensity units/mm2 (Day 1: 1256 ± 145, Day 2: 2678 ± 156, Day 3: 2287 ± 189, Day 4: 4018 ± 178) * Statistically significant when compared to the production on first day p < 0.05. **(B) Changes in the distribution of β-catenin in HUVECs **HUVECs were maintained in culture in presence of curcumin for (10 μM) for different time intervals as above. The cells were harvested and nuclear and cytosol were isolated. Protein equivalent amount of the cytosolic and nuclear fractions were subjected to SDS PAGE and western blot analysis with anti β-catenin antibody as described in the text. Values for control in intensity units/mm2/mg protein (Nucleus Day 1: 1877 ±, 145 Day 2: 1687 ± 102, Day 3: 987 ± 89, Day 4: 417 ± 96 and Cytosol Day 1: 0, Day 2: 0, Day 3: 887 ± 69, Day 4: 1567 ± 58). Lane1-4 1^st ^-4^th ^day (inset). The intensity of the immunoblotted bands were measured and expressed in intensity units/mm^2^/mg protein (**a**). Percentage distribution of β-catenin in the nucleus and cytosol was calculated and expressed in the percentage bar diagram (**b**). Values for control (Nucleus Day 1: 100, Day 2: 100, Day 3: 52, Day 4: 21 and Cytosol Day 1: 0, Day 2: 0, Day 3: 48, Day 4: 79). * Statistically significant when compared to their relative levels in nucleus and cytosol on first two days p < 0.05.

Inorder to further examine the relation between the production of cell-adhesion molecules and MMPs by ECs during angiogenesis, changes in cadherin and catenin with the progression of culture were studied in cells treated with curcumin under serum free conditions. The results are given in Figure 7A. There was significant increase in the amount of VE-cadherin with the progression of culture which on the fourth day was about 4 fold more than that on the first day. RT-PCR analysis also showed that the level of mRNA of VE-cadherin in curcumin treated cells progressively increased with the progression of culture (data not given).

Analysis of the relative levels of β-catenin in the nucleus and cytoplasm of the cells treated with curcumin showed relatively higher levels of this protein in the nucleus during initial stages (upto 48 hrs) which declined thereafter concomitantly with significant increase in its levels in the cytosol during the later stages of culture (Figure 7B). In cells treated with curcumin only about 25% of the total β-catenin was present in the nucleus on the third day of culture. Immunocytochemical analysis of β-catenin in HUVECs maintained in culture for 24 hrs and 120 hrs further confirmed the presence of β-catenin in nucleus in ECs during initial stages (24 hrs) and their localization at the cell contact sites during later stages of culture (120 hrs) when ECs formed extensive cell-cell contact and capillary network-like structures (Additional File 1, Figure S1 and Additional File 2, Figure S2).

### Changes in the tyrosine and serine phosphorylation of β-catenin

**Figure 8:**
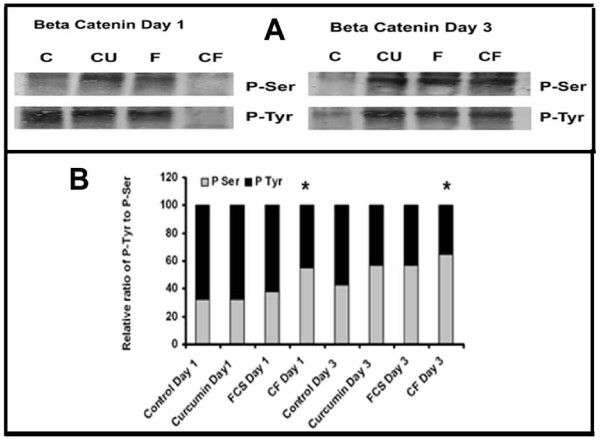
**Phosphorylation of β-catenin**. **(A) **Cell lysates equivalent to same amount of β-catenin, from cells treated with curcumin (10 μM) in the absence and presence of serum for different time intervals were treated with anti-β-catenin antibody. The immunoprecipitated β-catenin was electrophoresed and immunoblotted with anti-Phospho-Tyr and Phospho-Ser antibody (C control, Cu curcumin, FCS (FCS control), CF (Curcumin + FCS). **(B) **The intensity of bands were quantitated and the extent of phosphorylation was expressed as percentage bar diagram. Values given are average of quarduplicate experiments. * Statistically significant when compared to the relative ratio in FCS control p < 0.05.

In order to understand the phosphorylation status of β-catenin, the trigger that directs the nuclear translocation of cytosolic β-catenin, it was immunoprecipitated and probed with specific phospho-antibody and the results are shown in Figure 8.

The changes in the phosphorylation of tyrosine and serine in β-catenin in cells treated with curcumin in serum free and serum supplemented conditions for different periods were also studied. The extent of tyrosine phosphorylation of β-catenin on 3^rd ^day in culture was significantly less than that on the first day and that of serine phosphorylation was more on third day than the first day in culture in serum free condition. Since there was difference in the extent of phosphorylation among different treatments, the intensity of each was expressed as percentage of the sum of the intensities of bands corresponding to P-Tyr and P-Ser. The relative levels of tyrosine phosphorylation to serine phosphorylation of β-catenin was high during the initial stages of culture which decreased as the level of serine phosphorylation increased during later stages of culture in control and curcumin treated cells. But the ratio of tyrosine phosphorylation to serine phosphorylation of β-catenin was observed to be 1:1 in cells treated with curcumin in presence of serum which remained unaffected throughout the culture.

### Effect of Lithium on MMP production by HUVECs in culture

**Figure 9:**
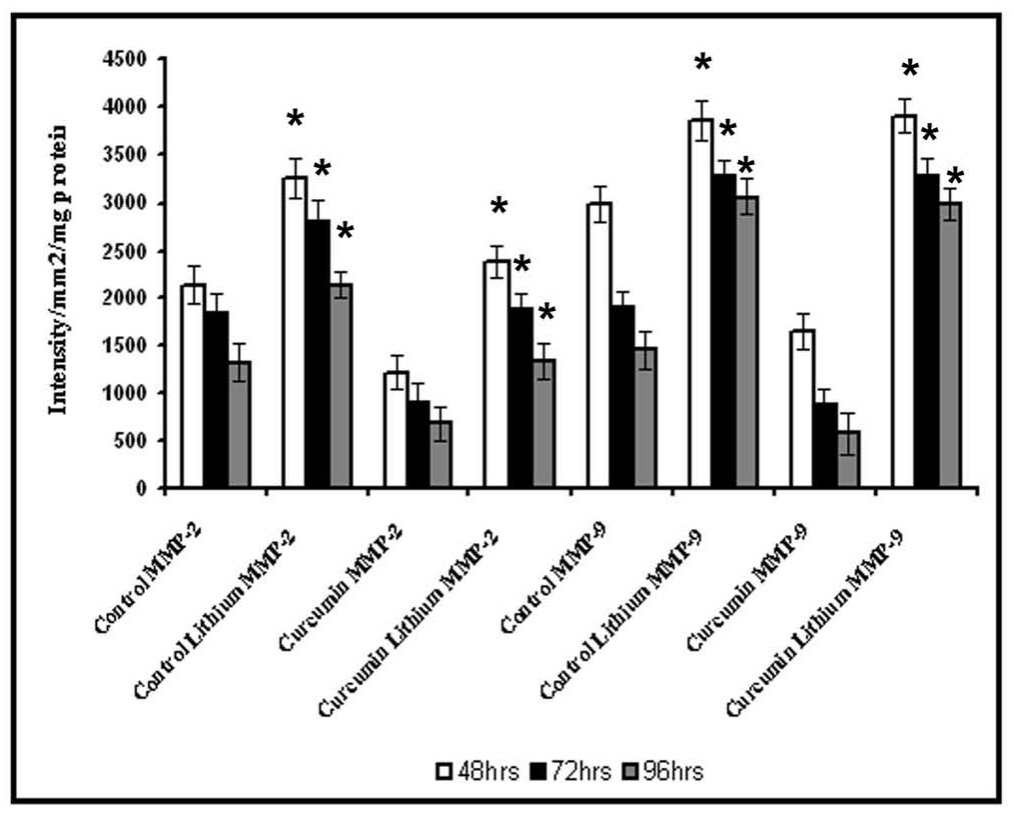
**Effect of lithium chloride on the activity of MMP-2 and MMP-9**. HUVECs were maintained in culture with 10 μM curcumin for 48, 72 and 96 hrs as described in text with and without GSK-3 inhibitor lithium chloride 1 mM. Medium was removed and the activities of MMP-2 and MMP-9 were determined by gelatin zymography. The intensity of MMP-9 and MMP-2 were measured and expressed in intensity units/mm^2^/mg protein. * Statistically significant when compared to untreated control p < 0.05.

To examine whether the activation of β-catenin is associated with the changes in MMPs, the effect of inhibition of the glycogen synthase kinase-3 (GSK-3), an enzyme that catalyses serine phosphorylation of β-catenin by lithium on the activity of MMPs was analysed. In cells treated with Li, no downregulation of MMPs was observed and there was significant increase in the activity of both MMP-2 and MMP-9 when compared to the respective control (Figure 9). In cells treated with curcumin along with lithium, the effect of curcumin was reversed and significant increase in the activities of both MMP-2 and MMP-9 was observed when compared with the cells treated with curcumin alone. These results suggest that inhibition of GSK-3 which catalyses serine phosphorylation of certain proteins including β-catenin results in reversal of the effect of curcumin on MMP production. The viability of the cells were not affected on treatment with lithium, but the extent of grouping was less and the angiogenic transition was delayed by Li in both controls and curcumin treated cells.

## Discussion

The results presented above indicate the regulation of the production of matrix metalloproteinases, MMP-2 and MMP-9 by endothelial cells during angiogenesis. There was significant decrease in the production and secretion of MMP-2 and MMP-9 with the progression of culture when grouping of cells and tubular network-like structure developed. Development of such structures involves extensive cell-cell contact formation which correlates with the expression of endothelial cell markers like CD 31, ICAM-1 and E-selectin that mediates cell adhesion and biochemical markers of angiogenesis such as VEGFR-2, FGFR-1 and VEGF [12,20,21]; thus a reciprocal relation between the expression of markers of angiogenesis, cell adhesion molecules and MMPs is evident.

Relation between production of MMP-2 and MMP-9 and angiogenic transition of ECs was also evident from the results of the experiments on MMP production by ECs treated with substances such as ursolic acid and curcumin that affect angiogenesis. Angiogenic effect of ursolic acid was shown by upregulation of the expression of angiogenic markers ICAM-1 and angiogenic factors like VEGF and fibroblast growth factor-2 (FGF-2) by ECs [20]. Opposing effects of curcumin on angiogenesis is shown using different model systems and the proangiogenic effect is mediated through VEGF and PI3K-Akt pathway [21]. Treatment of endothelial cells with these substances which promoted angiogenic transition of ECs caused downregulation of MMP-2 and MMP-9. But such downregulation of MMPs was not produced by aspirin which inhibited angiogenesis and reduced the production of cell adhesion molecules [30,31] that promote cell-cell contact formation.

The downregulation of MMPs minimizes peri-cellular proteolysis and facilitates the cell-cell/cell-matrix interactions which are vital for the maturation and stabilization of neo-vessels. It appears to be endothelial cell-specific and is related to the angiogenic transition associated with the formation of cell-cell contact. Other cell systems such as monoctye/macrophage [32], hepatic stellate cells [33], hepatocytes [34] and cell lines [35-37] in culture produced and secreted MMPs which increased with the progression of the culture. The effect of curcumin on ECs also appears to be cell type specific as curcumin did not affect the basal level production of MMP-2 and MMP-9 by other cell systems such as monocytes [38]. It caused significant inhibition in the production and secretion of MMP-2 and MMP-9 by activated monocytes [38] and cancer cells by inhibiting the pro-inflammatory transcription factors NFκB and AP-1 [39,40]. Further, adhesion proteins such as laminin and fibronectin, which promoted cell-cell contact and capillary tube-like structure formation, also caused downregulation of MMPs that was synchronous with the formation of cell-cell contact (Unpublished data). The activity and levels of protein and mRNA of MMP-2 and MMP-9 significantly decreased with the progression of the culture indicating a downregulation of the expression of the MMP genes. This does not appear to be a general effect as several other molecules such as CD31, E-selectin, ICAM-1 and VE-cadherin increased with the progression of culture. Although there was no significant difference in the activity of MMP-2 and MMP-9 during 24-48 hrs, there was about 10-15% increase in the level of these proteins determined by ELISA on the second day (Figure 1 and 2). It is not clear whether this was due to the difference in the extent of activation of the proenzyme or due to other factors. The effect of endogenous inhibitors of MMPs namely tissue inhibitor of metalloproteinases (TIMPs) which increased under similar conditions [6] could not be excluded.

During the initial stages when cells remained mostly as individual ones, there was more MMP-2 and MMP-9 and less VE-cadherin and at later stages when grouping of cells and network-like structures developed, increase in VE-cadherin and decrease in MMP-2 and MMP-9 production were observed. Further, curcumin that accelerated angiogenic phenotype in HUVECs under serum free conditions caused a significant upregulation of VE-cadherin and downregulation of MMPs. Although direct proof relating cell-cell contact formation and increase in the levels and distribution of VE-cadherin is not provided in our present study, there are reports showing change in cell-cell contact with change in VE-cadherin expression [41,42]. It has been reported that during the initial stages of angiogenesis, the levels of VE-cadherin remained low due to increased endocytosis maintaining a very low level of this protein on the surface of the ECs to facilitate their migration and proliferation [43].

Further investigations to understand the signaling pathways downstream of VE-cadherin suggested the involvement of β-catenin. There was a relatively high level of β-catenin in the nucleus of ECs in culture during initial stages and decrease in its levels in the nucleus associated with an increase in the cytosol during later stages. Apart from contributing to stabilise cell-cell contact through cadherin-catenin association, the translocation of catenin can result in changes in the expression of catenin responsive genes. The activity of β-catenin is regulated by the phosphorylation status of this protein. Our results on increased tyrosine phosphorylation and relatively higher levels of β-catenin in the nucleus during initial stages (upto 48 hrs) and relative increase in serine phosphorylation and decreased nuclear distribution at later stages are consistent with the reported role of phosphorylation of β-catenin in its translocation from cytosol to nucleus [44,45]. Further, higher levels of VE-cadherin observed during later stages of culture may limit the β-catenin transcriptional activity due to increase in binding to VE-cadherin [46].

There was correlation between the distribution of β-catenin in the nucleus and the expression of MMP-2 and MMP-9. As β-catenin in the nucleus decreased with the progression of the culture, the expression of MMPs also decreased. Such a down regulation of MMPs was not observed on treatment with Li which caused increase in the nuclear distribution of β-catenin. The down regulation of MMP-2 and MMP-9 caused by curcumin was also reversed by Li. The effect of Li on the distribution of β-catenin may be related to its phoshorylation status as Li is known to inhibit GSK-3 which catalyses phosphorylation of serine in catenin. Similar results were also obtained on treatment of cells with SB216763 a specific inhibitor of GSK-3 (data not shown). However, the possibility of these substances acting on other intracellular pathways cannot be excluded. Although these results show a correlation between phosphorylation status and nuclear distribution of β-catenin and the levels of MMPs it is not clear whether the expression of MMP-2 and MMP-9 in HUVECs is dependent on VE cadherin-β-catenin signaling. MMP genes are under the influence of the transcriptional activity of β-catenin as β-catenin responsive elements are reported to be present in the MMP genes [14,47]. Although MMP-2 lacks these transcriptional control elements, it has been reported to be regulated by cadherin in epithelial cells probably by a catenin independent mechanism [48,49]. Consistent with this, we also observed downregulation of MMP-2 when the levels of VE-cadherin increased. The possibility of VE-cadherin modulating MMP-2 and MMP-9 expression through β-catenin-independent mechanism cannot be excluded.

Although reciprocal changes in the expression of MMPs and VE-cadherin were observed in HUVECs in these different experiments where both pro-angiogenic and anti-angiogenic substances were tested, it is possible that these changes may as well be unrelated.

As MMP level decrease probably by other unknown mechanisms pericellular proteolysis is reduced resulting in the retention of VE-cadherin and other cell adhesion molecules promoting cell-cell contact formation. More direct experiments would be required to confirm this relationship between the expression of the cell adhesion molecule in angiogeneiss and modulation of MMP expression.

## Competing interests

The authors declare that they have no competing interests.

## Authors' contributions

Most of the experiments were performed by MSK. All the authors contributed to analysis and interpretation of data, discussion and preparation of the manuscript. All the authors read and approved the final manuscript.

## Supplementary Material

Additional file 1 **Immunocytochemical analysis of the expression and translocation of β-catenin in HUVECs maintained in culture for 24 hrs**. ***Figure S1 Expression and translocation of β-catenin in HUVECs: Day1 ***HUVECs were cultured in MCDB 131 medium and maintained in culture for 5 days. Cells were harvested at Day 1 (24 hrs) immunocytochemical analysis was performed to analyze the expression of β-catenin. Left panel Top to bottom (a) microphotograph of cells Day 1 (40 X) stained with β-catenin, (b) overlaid microphotograph of cells with nucleus stained **(Hoechst stain)**, (c) phase contrast image of cells Day 1. Right panel top to bottom, (a) microphotograph of cells Day 1 (100 X) stained with β-catenin, (b) overlaid image with nucleus stained (**Hoechst stain**) and (c) microphograph of cells with nucleus stained. Arrow heads to the β-catenin stained red, nucleus stained blue. *The β-catenin is located in nucleus of the cells during day 1 where the cells are in single spherical morphology and no cell-cell contact formation observed*. Click here for file

Additional file 2 **Immunocytochemical analysis of the expression and translocation of β-catenin in HUVECs maintained in culture for 120 hrs**. ***Figure S2 Expression and translocation of β-catenin in HUVECs: Day 5***HUVECs were cultured in MCDB 131 medium and maintained in culture for 5 days (120 hrs). Cells were harvested at Day 5, and immunocytochemical analysis was performed to analyze the expression of β-catenin. Left panel Top to bottom microphotograph of cells Day 5 (40 X) stained with β-catenin, (b) overlaid image of cells with nucleus stained (**Hoechst **stain) (c) phase contrast image and (d) microphotograph of cells with nucleus stained **(Hoechst stain)**. Right panel Top to bottom, (a) microphotograph of cells Day 5 (100 X) stained with β-catenin, (b) microphotograph of cells with nucleus stained and (c) overlaid microphotograph with nucleus stained (**Hoechst stain**). Arrow heads to the β-catenin stained red, nucleus stained blue. *HUVECs formed extensive cell-cell contact and capillary network like structure. The β-catenin is located at the cell contact sites*. Click here for file
