# Supplementary figures and images for: Changes in expression of VE-cadherin and MMPs in endothelial cells: Implications for angiogenesis
Source: Vasc Cell. 2011 Feb 14;3:6. doi: 10.1186/2045-824X-3-6 (PMC3045352; doi:10.1186/2045-824X-3-6)

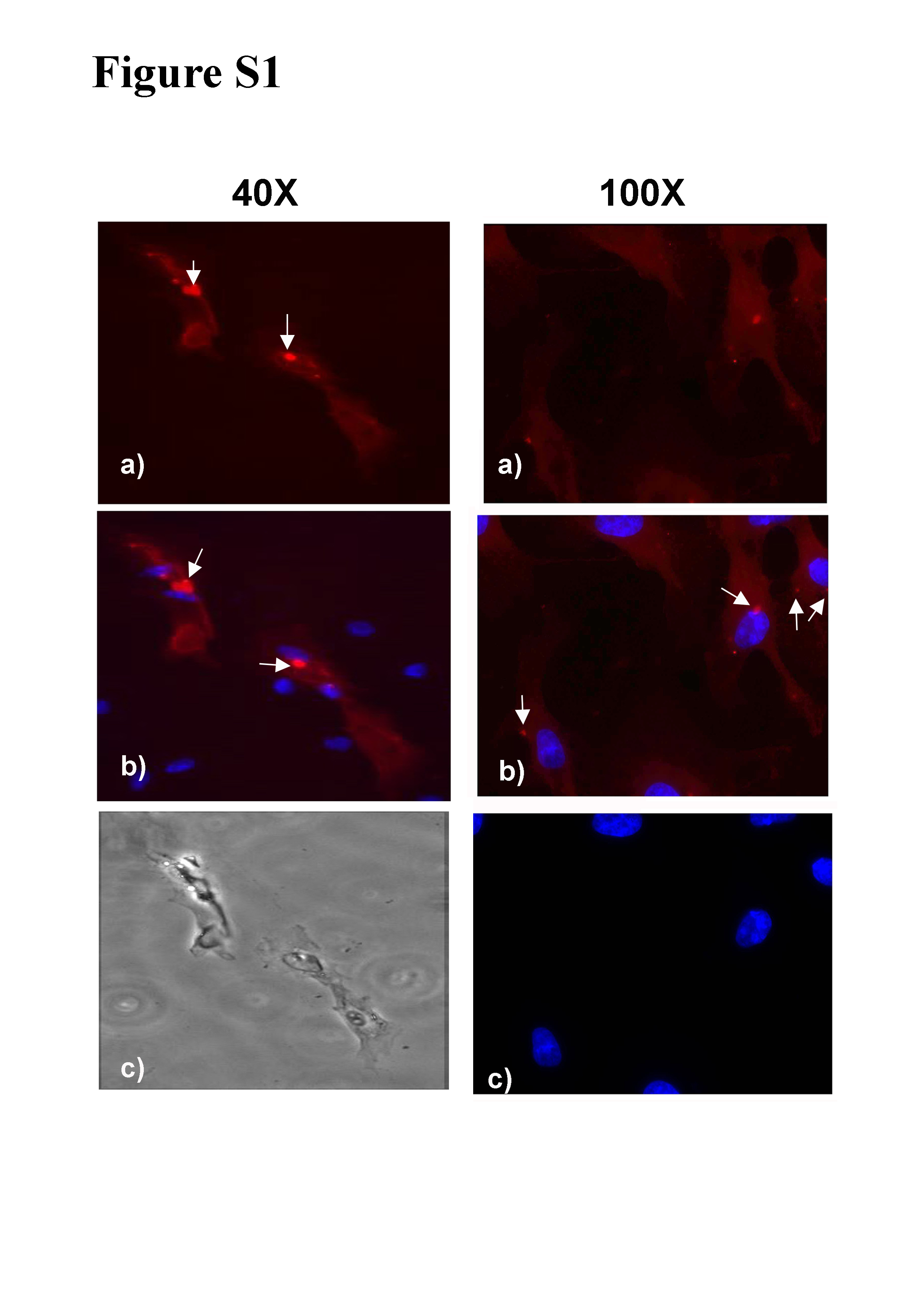

Supplement: Additional file 1 — Immunocytochemical analysis of the expression and translocation of β-catenin in HUVECs maintained in culture for 24 hrs. Figure S1 Expression and translocation of β-catenin in HUVECs: Day1 HUVECs were cultured in MCDB 131 medium and maintained in culture for 5 days. Cells were harvested at Day 1 (24 hrs) immunocytochemical analysis was performed to analyze the expression of β-catenin. Left panel Top to bottom (a) microphotograph of cells Day 1 (40 X) stained with β-catenin, (b) overlaid microphotograph of cells with nucleus stained (Hoechst stain), (c) phase contrast image of cells Day 1. Right panel top to bottom, (a) microphotograph of cells Day 1 (100 X) stained with β-catenin, (b) overlaid image with nucleus stained (Hoechst stain) and (c) microphograph of cells with nucleus stained. Arrow heads to the β-catenin stained red, nucleus stained blue. The β-catenin is located in nucleus of the cells during day 1 where the cells are in single spherical morphology and no cell-cell contact formation observed. [file 2045-824X-3-6-S1.TIFF]

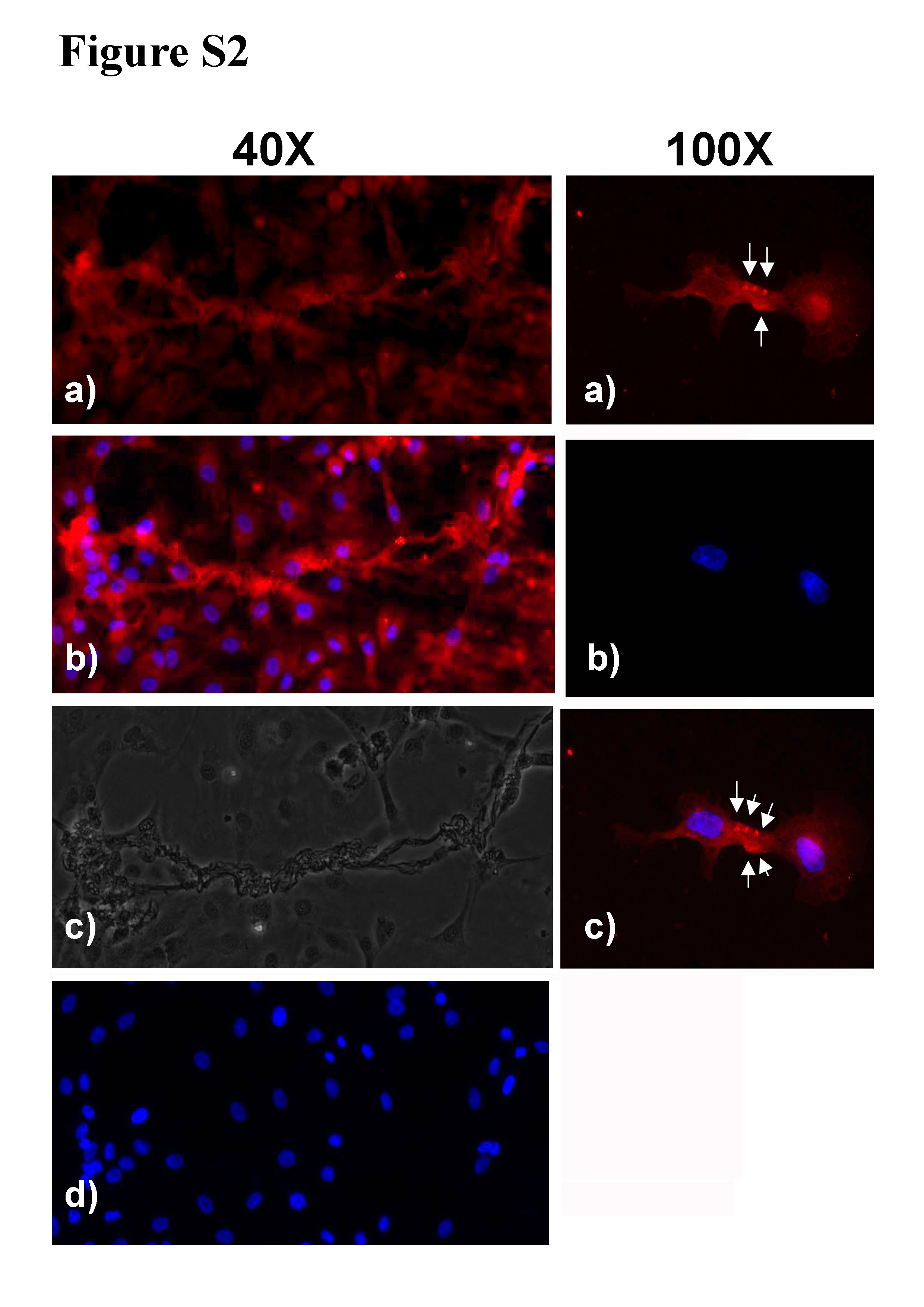

Supplement: Additional file 2 — Immunocytochemical analysis of the expression and translocation of β-catenin in HUVECs maintained in culture for 120 hrs. Figure S2 Expression and translocation of β-catenin in HUVECs: Day 5HUVECs were cultured in MCDB 131 medium and maintained in culture for 5 days (120 hrs). Cells were harvested at Day 5, and immunocytochemical analysis was performed to analyze the expression of β-catenin. Left panel Top to bottom microphotograph of cells Day 5 (40 X) stained with β-catenin, (b) overlaid image of cells with nucleus stained (Hoechst stain) (c) phase contrast image and (d) microphotograph of cells with nucleus stained (Hoechst stain). Right panel Top to bottom, (a) microphotograph of cells Day 5 (100 X) stained with β-catenin, (b) microphotograph of cells with nucleus stained and (c) overlaid microphotograph with nucleus stained (Hoechst stain). Arrow heads to the β-catenin stained red, nucleus stained blue. HUVECs formed extensive cell-cell contact and capillary network like structure. The β-catenin is located at the cell contact sites. [file 2045-824X-3-6-S2.TIFF]
